# Patient and therapist experiences of a self-management group programme for fatigue in primary care: a qualitative study

**DOI:** 10.1186/s12875-026-03450-y

**Published:** 2026-07-03

**Authors:** Sharon H. Gunnink-Wennink, Edith H.C. Cup, Yvonne Veenhuizen, Thomas J. Hoogeboom, Philip J. van der Wees, Nicoline B.M. Voet, Maud Graff

**Affiliations:** 1 Donders Institute for Brain, Cognition and Behaviour, Department of Rehabilitation, Radboud University Nijmegen Medical Centre, Nijmegen, Netherlands; 2 UMC Utrecht Brain Center, University Medical Center Utrecht, Utrecht University, Utrecht, Netherlands; 3 Department of Rehabilitation, Physiotherapy Science & Sports, Knowledge Centre Healthy and Sustainable Living, Research Group Innovation of Human Movement Care, HU University of Applied Sciences, Utrecht, Netherlands; 4 IQ Healthcare, Research Institute for Health Sciences, Radboud University Nijmegen Medical Centre, Nijmegen, Netherlands; 5 Rehabilitation Centre Klimmendaal, Arnhem, Netherlands; 6 Radboud University Nijmegen Medical Centre, Nijmegen, Netherlands

**Keywords:** Primary care, Self-management, Group-based intervention, Chronic neurological disorder, Fatigue

## Abstract

**Introduction:**

As health systems shift care from specialised to primary care settings, questions arise about implementing complex interventions such as self‑management programmes. This is especially relevant for the growing population with chronic neurological disorders requiring long‑term support in primary care. ‘Energetic’, an interdisciplinary self‑management group programme delivered by occupational therapists and physiotherapists, was originally developed in specialist rehabilitation to improve participation and functional endurance in people with neurological conditions and fatigue. Although effective in specialist care, little is known about how patients and therapists experience its delivery in primary care, where resources and infrastructure differ substantially.

**Purpose:**

This study examines how patients with chronic neurological conditions and fatigue, and their therapists, experience the implementation and delivery of the Energetic self‑management group programme in primary care.

**Methods:**

Energetic was implemented in primary care in three regions in the Netherlands. A qualitative study was conducted using semi‑structured interviews with 10 patients and seven therapists (three occupational therapists and four physiotherapists) from three primary care settings. Interviews were audio-recorded and transcribed verbatim. Participants were asked about their experiences with participating in the programme, its implementation and delivery in primary care, and perceived barriers and benefits. Data were analysed using an iterative, inductive thematic approach.

**Results:**

Three overarching themes were constructed from the data: 1) The content of the programme is considered comprehensive; 2) The programme is believed to strengthen self-management and particpation; and 3) The implementation is experienced to be challenging within the primary care healthcare system. Patients experienced participating in the programme as meaningful, reporting better self-management and participation. Therapists reported similar developments. Regarding implementation therapists described persistent difficulties related to recruitment, organisation, and financing, and noted that system constraints required administrative improvisation to deliver the programme as intended. Differences in perspectives were also apparent: e.g., patients valued access to sports facilities, whereas all therapists considered such facilities difficult to provide within primary care.

**Conclusion:**

Patients and therapists experienced the Energetic programme as meaningful and valuable in primary care practice. However, therapists reported problems in recruitment, reimbursement and administrative barriers that constrained the programme’s implementation. These findings illustrate how promising programmes can struggle to find their way into routine primary care, and therefore to patients living longer at home.

**Supplementary Information:**

The online version contains supplementary material available at 10.1186/s12875-026-03450-y.

## Introduction

There is evidence that structured fatigue self-management programmes for people with chronic neurological disorders can improve participation and functioning [1, 2]. Several interventions have been developed and evaluated, including programmes that combine physical activity, education, and behavioural strategies to support long-term fatigue management within rehabilitation and, increasingly, primary care contexts [3–5]. In primary care, however, such approaches are often delivered as single-component interventions, or embedded within broader self-management programmes for chronic conditions. Evidence for comprehensive fatigue-specific programmes remains limited and heterogeneous [6]. As a result, it remains unclear how these programmes are actually delivered and experienced in primary care. This is especially relevant given the growing number of individuals with chronic neurological disorders who require long-term support within primary care [7, 8]. As health systems increasingly shift long-term care from specialist to primary care settings [9, 10], understanding what enables or constrains delivery, and what patients and therapists perceive as valuable or burdensome, is essential for sustainable implementation.

This need is particularly urgent given the high prevalence and impact of fatigue among individuals with chronic neurological diseases, affecting more than half of patients and substantially limiting daily functioning and quality of life [11, 12]. Fatigue is a complex, multifactorial symptom, with diagnosis explaining only 11% of its severity [13]. In combination with physical and cognitive limitations, fatigue reduces physical activity and social participation, placing substantial emotional, financial, and social burdens on patients, their families, and their social networks [14]. Since no cure is currently available for most chronic neurological disorders, care is primarily focused on maintaining functional abilities and supporting participation in daily life. Effective fatigue management is therefore essential to improving quality of life for these individuals and their families.

To address this, a multidisciplinary group programme has been developed, which marks a shift from a disease-oriented approach to a patient- and problem-oriented approach [15]. This self-management intervention, called ‘Energetic’, has been designed to enhance social participation and functional endurance in individuals with chronic neurological conditions and fatigue, irrespective of the specific diagnosis [15]. By focusing on fatigue as a shared, multifactorial experience across conditions, this approach focuses on individuals’ experiences and daily life goals rather than diagnosis-specific pathways [16]. This is particularly relevant for symptoms like fatigue that transcend diagnostic categories [13]. It may enhance person-centred care and support self-management [17], but can be challenging to integrate within diagnosis-oriented healthcare systems [18].

Energetic, delivered by occupational and physiotherapists, integrates aerobic exercise training (AET), education on AET, energy conservation strategies [19, 20], and relapse prevention [1]. Energetic is delivered over a 4-month period, during which patients attend twice weekly sessions in the first 9 weeks and once weekly sessions in the final 7 weeks. Sessions are conducted face-to-face in small groups, with a minimum of 4 and a maximum of 8 patients per group. The programme has demonstrated significant improvements in physical and social functioning, with patients reporting better fatigue management and enhanced quality of life [1]. Given its success in specialized rehabilitation settings and in line with the broader shift toward primary care, there is a strong rationale for expanding the programme to primary care, where a broader population can potentially benefit from it.

Although Energetic has demonstrated effectiveness, a knowledge gap remains regarding how patients and therapists experience its implementation and delivery in primary care. This qualitative study therefore explores the experiences of patients with chronic neurological conditions and fatigue, as well as their therapists, regarding the implementation and delivery of the Energetic self-management group programme in primary care.

## Methods

### Qualitative approach and research paradigm

This study was designed as a qualitative study, aimed at exploring experiences with delivering the Energetic programme in primary care. Sampling prioritised diversity of perspectives across different primary care settings. This approach reflects real-world conditions in early implementation phases.

This study followed an interpretivist paradigm [21] to comprehensively explore patients’ and therapists’ experiences. Interpretivism is a philosophical approach that emphasizes understanding the subjective meanings and interpretations that individuals attach to their experiences. This approach assumes that reality is socially constructed, meaning that in our study the experiences and interpretations of patients and therapists are essential for understanding how programme delivery is experienced in primary care [22]. This methodological framework allows for a deeper understanding of how patients and therapists perceive and make sense of their experiences of participating in the programme within the primary care setting [23].

An iterative process of collecting and analysing data was used to develop an in‑depth understanding of patient and therapist experiences. Data were analysed using inductive thematic analysis, through which themes and patterns were constructed [24]. In addition to this, an interpretative approach was applied to explore the deeper meaning participants attributed to their experiences, with the researchers’ own reflections being integrated into the analysis [22]. Both methods complemented each other and provided structure and depth. Findings are reported accordance with the Consolidated Criteria for Reporting Qualitative Research (COREQ) guidelines [25].

### Context and participants

The programme was delivered at three locations across the Netherlands, with settings A and B implemented in the spring of 2024 and setting C in the spring of 2025. Individual semi-structured interviews were conducted with patients and therapists between June and August 2024 and in June 2025, either face-to-face or via video call, up to two months after completing the programme. The one-year difference between settings A and B and setting C, resulted from the postponed implementation of the programme in setting C, which was delayed due to an insufficient number of eligible patients, a practice relocation, and personal factors of therapists, such as a high workload. All patients and therapists who participated in the Energetic programme in primary care were approached to participate in the interview. Patients received an information letter, provided by their therapist and those who expressed interest were contacted by the researcher to answer any additional questions they might have. Written informed consent was obtained from all participants prior to the interviews, including permission to use anonymised quotations for dissemination and publication.

### Ethical issues pertaining to human subjects

The Radboud Ethics Committee (dossier number 2023–16802) determined that this study did not require review by the Medical Research Ethics Committee (METC) under the Dutch Medical Research Involving Human Subjects Act (WMO).

### Data collection methods

A semi-structured interview guide was developed by the research team, based on the constructs of the PRISM and RE-AIM frameworks, and was refined through discussion among the study team members [26, 27] (Annex B). To incorporate the interpretative approach, the interview guide was designed to probe not only for experiences but also for feelings, thoughts, and the meaning participants attributed to the Energetic programme. Open-ended, nondirective questions were used to encourage participants to reflect on their personal interpretations, thoughts and feelings. Two separate topic guides were developed: one for patients and one for therapists. The topics focused on how factors influence the programme’s reach in primary care, how it was delivered in practice, its perceived effects, and the extent to which it could be sustained [27]. Attention was also given to how the programme fit within existing organisational structures and the practical, organisational, and financial conditions influencing implementation [26]. The interview guide translated these topics into questions about participants’ overall experiences with the Energetic programme in primary care, reasons for participation, experienced barriers, and perceived benefits. Participants were also asked to reflect on how the programme affected their daily lives, emotional well‑being, independence, and ability to self‑manage, as well as to offer suggestions for potential programme adaptations. The interviews lasted about 45 min and ended when all topics had been discussed in depth. Interviews were conducted in the participants’ chosen environment, including face-to-face at their home, the primary care practice, or via video calling. All interviews were led by the first author, SGW, and were audio-recorded with the consent of the patients and therapists. After each interview, the interviewer documented reflective notes in a reflexive diary to capture initial impressions, contextual factors, and potential influences on data collection.

### Data analysis

Prior to the analysis, the audio-recorded interviews were transcribed, anonymized, and uploaded to Atlas.ti version 24.0.0 software (ATLAS.ti Scientific Software Development GmbH, Berlin, Germany). ATLAS.ti was used to support both the coding and analysis processes. We analysed all interview transcripts and documents using inductive reflexive thematic analysis, in which sub-themes and themes were actively constructed through an iterative and interpretative engagement with the data. This approach supported the generation of rich, nuanced insights [28]. In the first phase, the first author (SGW) assigned codes to the transcribed text fragments. The codes were discussed with two co-authors, each with a different background from the first author (EC and YV), and discrepancies were resolved through team discussions until consensus was reached. In the second phase, codes were clustered into preliminary sub-themes in order to generate patterns within the text fragments. The results of this second phase were also discussed between the first author and the two co-authors. In the final phase, overarching themes were constructed by grouping related sub-themes, and these were discussed between SGW, EC, and YV, and later within the research group (SGW, EC, YV, NV, PW, and MG) until consensus was reached.

### Characteristics and reflexivity of the research team

The first author (SGW), a primary care physiotherapist trained in qualitative research, conducted all interviews. Although she worked at one of the participating practices, she was not involved in delivering the programme, and she had no prior relationship with patient participants.

Her professional background likely facilitated rapport and open communication, but may also have influenced data collection through shared assumptions or professional norms. Reflexivity was therefore embedded throughout the study, including regular team discussions to examine interpretations, address potential biases, and ensure transparency.

The research team consisted of allied health and medical researchers with expertise in qualitative and implementation research. Two occupational therapists (EC, YV) combined clinical practice and research and were involved in the development and earlier evaluation of Energetic. Three senior researchers in occupational therapy (MG), allied health care (PW), and physiotherapy (TH) contributed expertise in implementation, evaluation, and outcomes research. A rehabilitation physician (NV) provided expertise in exercise interventions for neuromuscular diseases and connected the study to the broader professional community.

### Techniques to enhance trustworthiness

Credibility and trustworthiness of the interviews were enhanced by the timing of data collection, with a maximum of two months between the lived experiences and the interview, providing a good balance between obtaining detailed recollections and minimizing distortion due to time. However, potential influences such as recall bias were also considered, assuming that participants’ experiences were still fresh in their memory, but that some details might have been distorted [29]. Member checking was applied by sharing preliminary interpretations with participants, allowing them to confirm accuracy and correct misunderstandings [23]. Method triangulation was achieved by comparing patient and therapist perspectives and investigator triangulation by involving different researchers from the research team in the data analysis, resulting in a rich variety of perspectives and interpretations [30]. The consensus meeting with the multidisciplinary research team further improved the transparency and thoroughness of the data analysis. Following this, the preliminary findings were discussed by external experts (including patient associations and professional associations). By sharing the findings and inviting expert reflections, we validated our interpretations, identified potential blind spots, and ensured alignment with patient and professional perspectives, thereby reducing potential bias and strengthening the overall trustworthiness of the research. Detailed descriptions of patients, practitioners, and contexts were provided to enable assessment of transferability to comparable settings and to inform potential application in other healthcare contexts and populations.

## Findings

Patients with various chronic neurological conditions had participated in the Energetic programme, having been enrolled by the therapists. Of the 16 patients enrolled in the programme, one patient stopped in the first week due to hospital admission. Ten patients consented to participate in the interview, while five declined across the three primary care settings. For those patients who did not participate, reasons were not documented. Characteristics of the patients are presented in Table 1. Available demographic information suggested no obvious differences between patients who declined and those who participated in the interviews. From each setting, one physiotherapist and one occupational therapist participated, and in one setting two physiotherapists participated in the programme. The physiotherapists had a mean of 14.5 years of experience with neurological patients (range 1–35), while the occupational therapists had a mean of 14.3 years of experience (range 5–20).

**Table 1:** Patient characteristics

Number of participants	Total: 10
Setting 1	7
Setting 2	2
Setting 3	1
Sex	
men	3 (33%)
Age in years - mean (range)	55 (33-77)
Education level	
High	5
Middle	5
Low	0
Paid employment	4
Living with partner	9
Diagnosis	
Multiple Sclerosis	2
Parkinson’s Disease	3
Cerebral Vascular Accident	1
Small fibre neuropathy	1
Muscular dystrophy	1
Polyneuropathy	1
Rheumatoid Arthritis	1

**Fig. 1:**
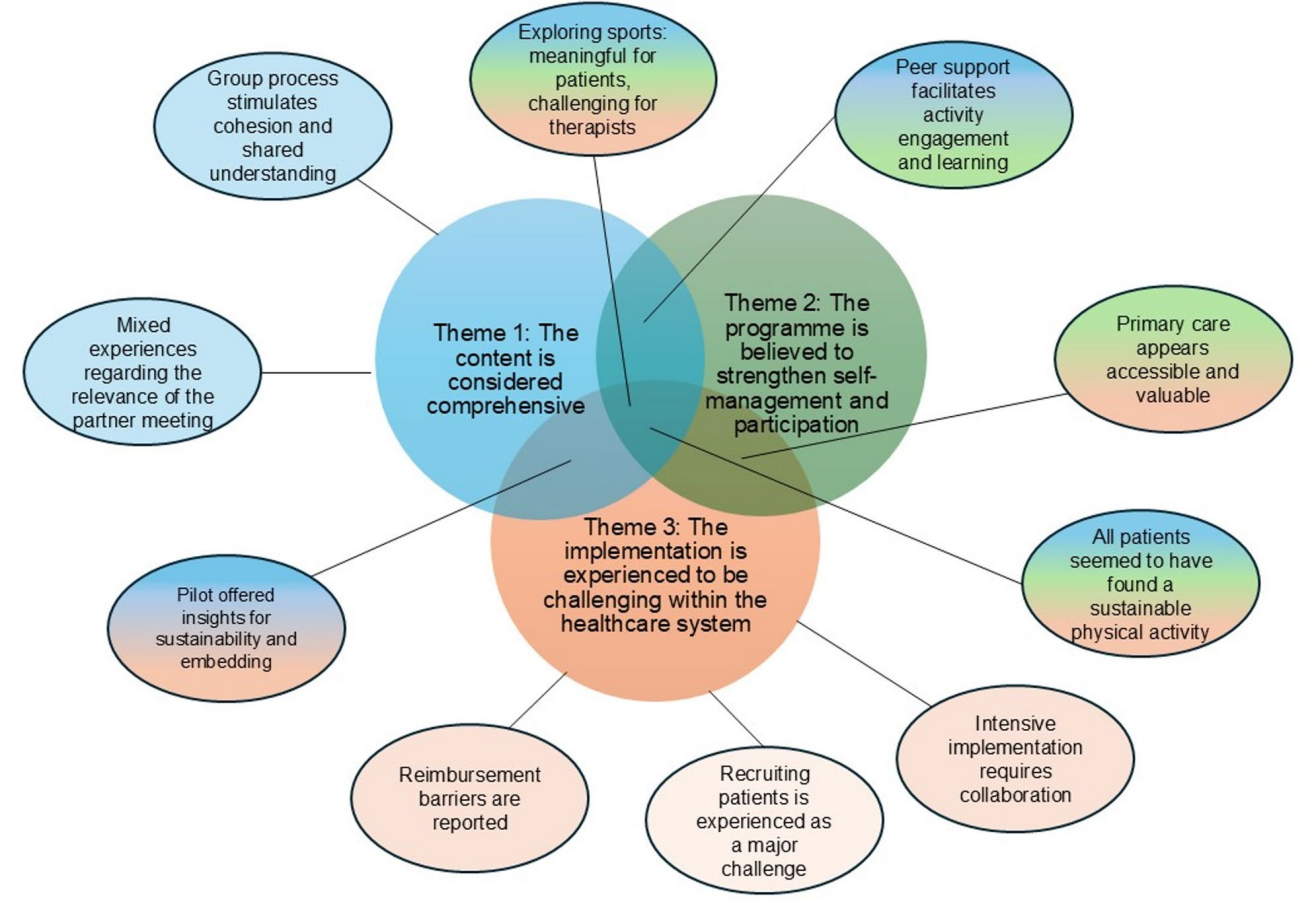
Themes, subthemes and their overlap (as shown by the colour transitions) regarding the experiences of therapists and patients with the Energetic programme in primary care

Through analysis of the interviews, three overarching themes were constructed: 1) The content of the programme is considered comprehensive; 2) The programme is believed to strengthen self-management and particpation; and 3) The implementation is experienced to be challenging within the primary care healthcare system. These themes are interrelated, overlapping and mutually influential. They are further divided into 10 subthemes that combine the perspectives of both patients and therapists (see Fig. 1).

The following sections describe the subthemes in detail, organized under the main themes to which they most strongly relate, while acknowledging that several subthemes span multiple domains. To protect the anonymity, patients are assigned numerical identifiers. The patient number and the setting in which the patient participated in the programme are indicated in brackets after each quotation. For quotations from therapists, the profession and the setting in which they work are provided in brackets. For reasons for anonimity of the therapists, the setting is indicated with a capital.

### Theme 1: the content of the programme is considered comprehensive

Therapists and patients described the programme as personally relevant and comprehensive, noting that the four integrated modules strengthened their self-management and provided a clear sense of direction and purpose. Therapists valued the well-developed educational materials and practical tools, which offered a solid foundation, contextual understanding and background knowledge for guiding patients. The combination of physical and educational elements was considered valuable by both patients and therapists and helped patients translate theoretical concepts into practical experiences.

“*There was a good balance between theoretical explanation and applying it yourself. During the sports sessions*,* that worked especially well*,* you start exercising and immediately learn how to manage your limits.” (*patient 3, setting B*).*

“In a short amount of time, you cover many different topics that are all interconnected.” (OT, setting C).

Patients appreciated the personalised approach, offering space for personal input and individual concerns, emphasising that they felt recognised as individuals rather than defined by their diagnosis. Although patients experienced the Energetic programme initially as intensive, they reported that they gradually learned to set priorities and integrate the programme into their daily routines. The ability to persist through the initial phase also pointed to the importance of patients’ intrinsic motivation. Therapists emphasised that completing the full programme was necessary to achieve behavioural change, highlighting that change is perceived as a gradual process in which repetition, reflection, and practice are essential for internalising new patterns.

*“You don’t just break through thought patterns overnight*,* they need the whole programme to reach new insights.” (OT*,* setting B)*.

*“At the beginning it was very intensive to be present for a whole day*,* but this improved and eventually I was no longer so tired at the end of the day. You just have to get through it.” (patient 9*,* setting A)*.

The group process stimulates cohesion and shared understanding. Both therapists and patients reported a positive and safe atmosphere, mutual recognition, and a strong sense of cohesion as key success factors of the programme. Functioning as a group appeared to strengthen motivation and contribute to a collective sense of responsibility.

*“As a group we felt that we had to make sure we stayed well for as long as possible.” (patient 7*,* Setting B).*

Despite diagnostic diversity, patients noted that shared fatigue created an immediate sense of understanding that reduced the need for explanation and lowered the threshold for connection. This implicit understanding strengthened the sense of belonging and made it easier to discuss personal limitation. At the same time, the variation in diagnoses provided new insights; seeing their own challenges reflected in others while also gaining different perspectives reinforced the idea that their struggles were part of a broader pattern rather than isolated experiences.

*“It doesn’t matter if I am compared to a patient with MS or Parkinson’s. They are all neurological conditions*,* and we are fatigued.” (patient 8*,* setting B).*

Both therapists and patients emphasized that the group setting accelerated the learning process by offering complementary viewpoints. Sharing experiences made theoretical concepts more concrete, applicable and personally meaningful.

*“Through the group process*,* theory is discussed*,* and it resonates more strongly. It is now much more conscious.”* (OT, setting B).

Peer support played a central role in encouraging activity, engagement and learning. Patients felt motivated to try new activities they would not attempt individually, and therapists observed that group motivation lowered thresholds for participation. Patients engaged in activities they previously avoided, suggesting that the group dynamic also helped challenge long standing fears or hesitations.

*“They motivate each other and can pull each other along. For example*,* people said they did not really want to go to badminton*,* but they would if everyone else went. On my own I would never get someone to participate in a new sport so quickly.” (OT*,* setting B).*

*“Because of everyone’s motivation and the programme*,* I started cycling again*,* something I had not dared to do for a long time.” (patient 6*,* setting B).*

Exploring sports at local sport clubs was experienced as meaningful for patients but challenging for therapists. Some sites already had connections with local trainers or sport clubs, while others had to build these networks from scratch. The involvement of a neighbourhood sports coach was valued but not available everywhere, indicating that the programme was better supported where such structures existed and required additional organisational investment where they did not. Therapists aimed to align sport activities with patient preferences, thereby reducing barriers by familiarising them with community sports environments. Patients consistently valued these lessons at local sport clubs, which helped them discover new activities.

*“Framerunning was an eye-opener. On my own*,* I would never have gone there.”* (patient 6, setting A).

In one setting, therapists perceived limited patient interest for exploring new sports, although a patient expressed disappointment about the absence of sports exploration.

*“There were no sessions to explore different sports. I really missed that.”* (patient 10, setting C).

This discrepancy suggested that therapists underestimated or misread patients’ interest, partly because decisions about external sports sessions were not made through shared decision making and that therapists’ own priorities and the extent to which they actively promoted these sessions, influenced whether available sports opportunities were used.

Experiences regarding the relevance of the partner meeting varied across settings. In some settings, patients noted that their partners or relatives felt insufficiently connected to the programme because they had not been involved from the beginning of the programme or were reluctant to share personal experiences.

*“The partners felt too distant from the programme and didn’t feel the need to talk about their private matters.”* (PT, setting B).

In other settings, therapists noted that patients’ partners found recognition in each other’s stories and valued the opportunity to exchange experiences. Patients also reported that the session was meaningful for their partners, who appreciated realising that their challenges were shared.

“*My partner also faced challenges*,* maybe not in the same way as other partners*,* but for him it’s important to know he’s not alone.”* (patient 9, setting A).

These differences reflected variation in group dynamics, facilitation style, and partners’ support needs. They also highlight the complexity of involving partners or close support persons in a patient‑centred programme.

### Theme 2: the programme is believed to strengthen self-management and participation

Through the programme, patients reported greater acceptance of their situation and gained new insights into their abilities and limitations. They became more aware of their energy balance and learned to plan and pace activities more effectively, reflecting a meaningful shift in how they related to their health challenges and suggesting that the programme supported a movement toward strengthened self‑management and participation. This shift was not merely practical but also emotional: patients reported increased self‑compassion, reduced performance pressure, and a renewed sense of autonomy.

*“I discovered which sports suit me well and I now have a clearer picture of my challenges*,* such as recognizing and accepting my limits in time. I do not need to be so performance oriented.”* (patient 5, setting A).

These skills also translated into behavioural changes in the work context. Patients described more effective communication about limitations, setting boundaries, and gradually building up their reintegration. They actively shaped their environment rather than merely adapting to it.

*“I have learned to communicate about my fatigue to my employer. Now I take more breaks during the day*,* and I no longer need to go home after the morning.”* (patient 6, setting B).

Patients emphasised the importance of re‑engaging in society and participating in meaningful activities despite limitations, reflecting a shift from focusing on restrictions to reconnecting with what is possible. The programme appeared to support both practical skill development and a broader reorientation toward one’s life and future perspective.

*“That you actually move beyond your illness and do not remain stuck in what is no longer possible. Yes*,* that you can participate in society again. Doing things you enjoy*,* despite your limitations.”* (patient 2, setting A).

Therapists noted that patients became motivated to remain physically active and were able to identify activities that suited them and continue these independently. Patients themselves reported being able to resume activity after setbacks, reflecting growing resilience and a strengthened belief in their own capacity to regulate activity levels.

*“I got COVID*,* but I knew how to pick it up again myself.”* (patient 9, setting A).

Ultimately, many patients reported that they had found a form of physical activity that seemed sustainable for them and that they felt able to perform responsibly without relying on physiotherapy. According to therapists, the educational component played a key role in this transition by helping patients judge appropriate exertion and trust their own decision making.

*“At least two patients initially said that they always needed physiotherapy to get the right exercise. They felt that without it they would do too little and deteriorate. The educational component of the Energetic programme was very valuable in teaching them what constitutes sufficient and appropriate activity*,* enabling them to judge for themselves when they had done enough and reducing their perceived need for physiotherapy support.”* (OT, setting A).

### Theme 3: the implementation is experienced to be challenging within the healthcare system

Primary care is experienced as accessible and valuable by patients and therapists. Therapists reported that implementing the programme in primary care offered advantages over rehabilitation centres or hospitals. Patients emphasised that the proximity and accessible atmosphere reduced the demands of participation, lowering logistical and energy-related barriers and enabling more consistent engagement, which may be particularly relevant for individuals with fatigue‑related limitations.

*“It was very pleasant that it was nearby; otherwise*,* I would not have gone to the hospital*,* that would have been too intensive.”* (patient 8, setting B).

The group‑based structure and the neurological‑ and fatigue‑specific modules distinguished the programme from regular primary care and were perceived as a valuable complement to standard services, providing specialist expertise within an accessible environment and helping to bridge the gap between routine primary care and more specialised rehabilitation care.

*“There is a lot of expertise in the programme; it does not have to be in the hospital.”* (patient 3, setting B).

Practising in familiar environments fostered continuity and long-term adherence by lowering the threshold for joining local sports clubs.

*“I now go to table tennis once a week; we also went there with Energetic.”(patient 9, setting A). “After the introductory lesson I continued with Nordic walking*,* where we also had a trial session*.” (patient 4, setting B).

Implementation of the Energetic programme in primary care experienced as intensive. Therapists explained that planning, logistics, and session preparation require substantial time and effort. These demands exceeded typical primary care structures and reflected both structural limitations and the high level of professional commitment involved. Despite the workload, therapists considered the programme valuable, implying that the perceived burden does not diminish its relevance but rather highlights the need for adequate support to ensure sustainable integration into primary care.

*“I did a lot in my free time; I spent about an hour preparing for each session.”* (OT, setting B).

Interdisciplinary collaboration was viewed as essential but not always easy to achieve. Teams used multiple communication channels. Communication through WhatsApp messaging was primarily employed for low-threshold organizational matters, the secure Siilo application ensured the safe communication regarding patient information, and shared email was used for patient and external contacts. Regular team check-ins reinforced alignment and co-location facilitated informal and spontaneous consultations. These strategies reflected local adaptations to the complexity of interdisciplinary care and highlighted the need for structural support.

*“Because we see each other often*,* it’s easy to have a quick hallway conversation.”* (OT, setting A).

In settings without such informal contact, therapists perceived collaboration as limited. This suggested that, beyond formal meeting structures, informal encounters played a key role by enabling frequent and accessible exchanges that fostered mutual trust, information sharing, and a shared treatment vision.

*“At the very beginning*,* before the programme started*,* we met once or twice to see if we could run it. After that*,* we mostly coordinated by phone or Teams. I missed that collaboration.” (OT*,* setting C).*

Recruiting patients was a major challenge according to the therapists. Despite efforts using flyers and outreach to referring physicians such as general practitioners, neurologists, and rehabilitation specialists, responses were limited.

*“We contacted neurologists*,* rehabilitation specialists*,* and general practitioners*,* but we didn’t receive any response.”* (OT, setting C).

Most patients were recruited from within the practice, often through existing therapeutic relationships, underscoring the influence of trust on willingness to participate. These challenges pointed to the need for clearer referral pathways, improved communication with referrers, and shared responsibility across disciplines to support programme uptake.

*“Seven out of eight patients came from our own practice population; they were already in treatment and were referred by colleagues.”* (PT, setting B).

Reimbursement barriers and administrative obstacles were the main issues that hindered sustainable implementation of the Energetic programme in primary care. They emphasised that the current Dutch reimbursement system, designed for individual, hourly treatment, does not align with the group-based and interdisciplinary nature of the programme. The current financing model incentivizes individual productivity over interdisciplinary group-based care. Therapists also indicated that a significant portion of programme-related work, including meetings, preparation, attendance during sports activities, and administrative tasks, fell outside reimbursable direct contact time.

Differences in reimbursement rules between different allied health professionals in primary care, combined with treatment indexes for physiotherapists and limits on billable contacts for occupational therapists, further restricted their ability to claim costs from insurers. Therapists noted that they felt forced to engage in administrative creativity in order to deliver the programme as intended within existing system constraints.

*“The treatment index becomes an issue. Your average number of treatments suddenly increases due to the programme. That can cause problems with the insurer.”* (PT, setting A).

*“Normally I can’t declare twice in one week for the same client*,* so that causes friction with the insurer.”* (OT, setting A).

These constraints also reduced flexibility, as occupational therapists often had to use all available hours for a single patient to complete the programme, leaving no room for follow-up care or the need for additional questions for occupational therapy. Collectively, these barriers highlighted that current funding mechanisms offer limited scope for interdisciplinary, group-based care. Therapists expressed a clear need for a dedicated reimbursement code to legitimise the programme and reduce administrative friction.

*“All time related to the programme and recurring activities should be calculated*,* and there should be a fixed fee for the programme as a whole*,* not having to declare each treatment separately. That should include organization and meeting time as well.”* (PT, setting B).

Pilot implementation offered insights for sustainability and embedding. Therapists described the pilot as both a feasibility test and a learning process that clarified roles, expectations, and communication patterns. Familiarity with the programme reduced preparation time and strengthened collaboration, suggesting that subsequent implementation cycles may be more cost-efficient.

*“I think we did it right the first time and implemented all the necessary adjustments for our setting. Now we have a kind of manual that we can*,* in principle*,* use again next year.”* (PT, setting A).

However, therapists also highlighted the need for greater programme visibility to support recruitment and financial feasibility remained dependent on enrolling a sufficient number of participants.

## Discussion

This study shows that the Energetic programme is experienced as meaningful, comprehensive, and personally relevant by both patients and therapists in primary care. Participants reported improvements in self-management, participation, and sustained physical activity, indicating that the intervention content is well aligned with the needs of people with chronic neurological conditions and fatigue. At the same time, our findings reveal that the main challenges do not lie in the programme itself, but at the system level. Organisational structures and reimbursement mechanisms in Dutch primary care are insufficiently aligned with interdisciplinary, group-based interventions. This mismatch between perceived value and structural support illustrates a broader tension within ongoing healthcare reforms aimed at delivering appropriate care closer to home.

### Transdiagnostic value

A previous study demonstrated the effectiveness of Energetic in clinical settings for people with neuromuscular disorders and chronic fatigue [15]. Our findings showed that the programme was experienced as meaningful in primary care by patients and therapists from different neurological backgrounds. The positive experiences of the heterogeneous group confirm that the programme is a promising intervention for persons with fatigue who have a wide range of chronic (neurological) conditions. This aligns with the previous study [13] showing that only a small proportion of variance in fatigue is explained by diagnosis, while transdiagnostic factors such as reduced activity, self-efficacy and physical functioning play a larger role. Our results confirm that fatigue, as a multidimensional and transdiagnostic phenomenon, requires an interdisciplinary and integrated treatment approach [13, 31]. The heterogeneity of diagnoses was not a limitation but reinforced the transdiagnostic nature of fatigue management in routine primary care.

Group dynamics and peer support strongly supported engagement in new physical activities, consistent with studies showing that shared experiences enhance adherence and motivation [28]. Therapists noted better retention of information in a group setting, suggesting that Energetic may be more effective and efficient than usual primary care.

Our findings also indicate that skills such as communicating about limitations and pacing contribute to return to work and employment participation. This is consistent with studies that describe boundary setting as a crucial skill for work participation [32]. The transdiagnostic approach and heterogeneous group composition fit well with primary care, increase applicability, and may facilitate broader implementation.

### Implementation in primary care

Internationally, fatigue management and self-management programmes have been implemented in primary care [33, 34]. A distinguishing feature of Energetic is its explicit integration of physical activity and sport with energy management strategies. Participants valued the opportunity to explore different forms of physical activity, which helped them identify suitable exercise and re-engage in meaningful activities, thereby reducing avoidance behaviours. This aligns with literature on activity pacing, which emphasises maintaining engagement in meaningful activities while preventing inactivity [35].

Implementation in primary care also addressed limitations previously observed in clinical settings. Earlier evaluations noted difficulties in sustaining physical activity independently at home and expressed a preference for neighbourhood-based sports [5]. In this study, trial sessions with local sports providers lowered the threshold for ongoing participation, and many patients ultimately engaged in a sport or physical activity within their own community. Although the programme was experienced as intensive, involving physical, cognitive, and organisational demands, patients reported increased awareness of their energy management capacities and improved participation in daily life. Consistent with previous findings, participation was perceived to improve both performance and satisfaction in performing meaningful activities, while the primary care setting reduced practical burdens such as travel, which had previously been experienced as a stressor [5].

### Primary care challenges

The programme is a group intervention that simultaneously addresses attention to the individual. Although a manual exists, it is not a one-size-fits-all approach, and effective delivery in primary care requires therapists to develop competencies to prioritise individual needs within diverse groups. Initial experiences with group interventions varied among therapists but were not perceived as a limitation. Partner involvement added a dyadic layer to an already complex group intervention, raising questions about how relational self-management can be structurally embedded in primary care. While involving partners may enhance support and sustainability of behavioural change, it also increases organisational complexity, particularly in primary care where time, coordination, and reimbursement structures are already constrained [36–38].

Therapists in all settings highlighted the organisational intensity, the complexity of scheduling within their work routines, and challenges of offering diversity in sport lessons. Implementation was time-intensive and not reimbursed within the current Dutch primary care system, which does not support group‑based or interdisciplinary interventions, posing a barrier to sustainability. Similar challenges have been reported in other primary care programmes, such as integrated care for diabetes and COPD, where financing remains focused on individual consultations and collaboration is not structurally reimbursed [39, 40]. Policy adaptations, such as specific reimbursement codes or bundled payments, are therefore essential to enable sustainable implementation.

Our findings align with the broader policy movement toward “appropriate care in the right place”, which emphasizes shifting care from secondary to primary settings. This study illustrates that a group programme focusing on self-management and participation, can be delivered closer to patients’ daily lives, thereby improving accessibility, reducing pressure on specialized services, and contributing to sustainable care.

### Strengths and limitations

Strengths of this study include the integration of both patient and therapist perspectives, which enabled perspective triangulation and enhanced credibility [41]. Rich qualitative data, supported by illustrative quotations, further strengthened the findings [42, 43]. In addition, a member check contributed to the trustworthiness of the results by enabling participants to verify the accuracy of interpretations [41]. The iterative process of data collection and analysis enabled the refinement of themes and a deeper understanding of participants’ experiences [44]. The combination of inductive thematic analysis and an interpretative approach provided both structure and depth, thereby enhancing the validity of the findings [43].

Several limitations should also be acknowledged. Within an interpretivist paradigm, the findings are context specific and aim to provide transferability. Moreover, the results are closely tied to the Dutch primary care context, where healthcare organisation, financing structures, and the availability of local sports providers shape implementation experiences, potentially limiting applicability to other health systems. An additional article currently in preparation will further examine facilitators and barriers to implementation, clarifying which elements of the Energetic programme are transferable across settings and which depend on local structures [45]. Future research could also incorporate additional perspectives, such as those of informal caregivers, to provide broader and richer insights.

Recruitment challenges and variation in group composition highlighted the pragmatic realities of implementing group-based interventions in primary care. Although a minimum group size of four participants had been recommended, this was not achieved in one setting, where the group consisted of only two patients. Of these, one met the criterion of having a chronic neurological condition with fatigue, while the other had a chronic condition accompanied by fatigue complaints outside the neurological domain. To still examine whether the programme was applicable in this context, implementation proceeded despite the deviation from the recommended group size and inclusion criteria. Including a mixed population reflects routine primary care practice, where fatigue is addressed across diagnostic categories, and supports exploration of the programme’s experiential fit beyond specialist rehabilitation settings. Interestingly, both therapists and patients in this specific setting reported positive experiences with the programme content. It should be noted that the inclusion of patients outside the original neurological target group represents a deviation from the intended design of the programme. However, these experiences, even in a small and diagnostically heterogeneous group, suggest that the core components of the Energetic Programme may be relevant beyond strictly defined neurological populations.

Limitations include the relatively small sample size and the absence of systematic documentation of reasons for non-participation. In addition, participants with lower educational backgrounds were not represented in our sample. Such a group may experience different levels of health literacy and access to physical activity services, which could influence both participation in the programme and study inclusion. This also suggests the need to consider how accessibility and recruitment strategies shape participation and how individuals with lower educational backgrounds can be better reached. Furthermore, while many patients were recruited from the therapists’ own practice population, this reflects real world recruitment pathways in primary care rather than introducing systematic selection bias. The reported challenges in recruitment may also be related to the nature of the programme itself, as transdiagnostic, problem-oriented interventions may not align well with existing diagnosis-driven referral pathways in primary care. This could partly explain difficulties in identifying and referring eligible patients. Consistent patterns across themes suggest data saturation, despite the small sample size. The role of the researcher, who was herself active in primary care, can be both a strength, through shared language and understanding, and a limitation, as assumptions may have been made based on personal experience. This may have influenced the data analyses and interpretation. By involving a multidisciplinary research team with members outside primary care, providing external perspectives, this was addressed.

While implementation experiences are shaped by national health system characteristics, the identified mismatch between perceived value and structural support is likely to be relevant beyond the Dutch context. This suggests that international efforts to promote self-management in primary care should address financing and organisational conditions alongside intervention design.

### Implications for future research

Next steps for further implementing Energetic in primary care are to evaluate its effectiveness and cost-effectiveness in these settings. Although Energetic’s effectiveness in clinical contexts for neuromuscular disorders has been demonstrated, its effectiveness and cost-effectiveness in primary care for patients with progressive neurological conditions and fatigue remain to be evaluated. Future research should focus on the system-level conditions required for sustainable delivery of the Energetic programme in primary care, particularly reimbursement models that support group-based and interdisciplinary care, alongside evaluation of effectiveness and cost efficiency, ideally through mixed‑methods research. Long-term follow-up is needed to determine whether patients remain physically active and sustain participation. Further research should also identify which target groups and severity levels benefit most from implementation in primary care versus specialist rehabilitation setting. In addition, strengthening multidisciplinary team training and awareness may be essential to ensure timely referrals, particularly in light of the recruitment challenges identified in this study. Finally, detailed economic evaluations are required to inform policy, secure sustainable funding, and ensure broad availability. Such efforts are key to expanding reach, alleviating pressure on specialized services, and delivering appropriate care in the right place.

## Conclusion

This study shows that the Energetic programme aligns well with the needs of people with chronic neurological conditions and fatigue and is experienced as meaningful and valuable within primary care. Patients reported improved self‑management and participation, and therapists recognised its relevance for supporting daily functioning. Despite these positive experiences, the structural conditions needed for sustainable delivery are still lacking. Therapists encountered financial and organisational barriers that hinder long‑term implementation. The challenges therefore lie not in the programme’s acceptability or perceived effectiveness, but in the extent to which current reimbursement and organisational structures can accommodate group‑based, interdisciplinary self‑management programmes. These findings illustrate how even promising interventions may struggle to become embedded in routine primary care, limiting their reach to people living longer at home.

## Supplementary Information

Supplementary Material 1.

## Data Availability

The datasets analysed during the study are not publicly available to ensure participants’ identity and data confidentiality. The de-identified transcripts are available from the corresponding author on reasonable request.

## Abbreviations

PT: Physical Therapist
OT: Occupational Therapist
PRISM: Practical, Robust Implementation and Sustainability Model
RE-AIM: Reach, Effectiveness, Adoption, Implementation, Maintenance
