# Supplementary material for: Patient and therapist experiences of a self-management group programme for fatigue in primary care: a qualitative study
Source: BMC Prim Care. 2026 Jul 3;27:290. doi: 10.1186/s12875-026-03450-y (PMC13445733; doi:10.1186/s12875-026-03450-y)
Supplement: Supplementary file 1 [file 12875_2026_3450_MOESM1_ESM.docx]

**ANNEX**

A. Consolidated criteria for reporting qualitative studies (COREQ): 32-item checklist
B. Interview Guides

**Annex A**

**Consolidated criteria for reporting qualitative studies (COREQ): 32-item checklist**

Developed from:

Allison Tong, Peter Sainsbury, Jonathan Craig, Consolidated criteria for reporting qualitative research (COREQ): a 32-item checklist for interviews and focus groups, International Journal for Quality in Health Care, Volume 19, Issue 6, December 2007, Pages 349–357, <https://doi.org/10.1093/intqhc/mzm042>

| **Item No** | | **Guide Questions/Description** | **Reported on Page #** |  |  |
| --- | --- | --- | --- | --- | --- |
| **Domain 1: Research team and reflexivity** | | | |  |  |
| **Personal Characteristics** | | | |  |  |
| 1. Interviewer/ facilitator | | Which author/s conducted the interview or focus group? | Pg 7 |  |  |
| 2. Credentials | | What were the researcher’s credentials? E.g., PhD, MD | N/A |  |  |
| 3. Occupation | | What was their occupation at the time of the study? | Pg 8 |  |  |
| 4. Gender | | Was the researcher male or female? | Pg 8 |  |  |
| 5. Experience and training | | What experience or training did the researcher have? | Pg 8 |  |  |
| **Relationship with participants** | | | |  |  |
| 6. Relationship established | | Was a relationship established prior to study commencement? | Pg 8 |  |  |
| 7. Participant knowledge of the interviewer | | What did the participants know about the researcher? e.g. personal goals, reasons for doing the research? | Pg 8 |  |  |
| 8. Interviewer characteristics | | What characteristics were reported about the interviewer/facilitator? e.g. Bias, assumptions, reasons and interests in the research topic | Pg 8 |  |  |
|  | **Domain 2: study design** | | |  |  |
|  | **Theoretical framework** | | |  |  |
|  | 9. Methodological orientation and Theory | What methodological orientation was stated to underpin the study? e.g. grounded theory, discourse analysis, ethnography, phenomenology, content analysis | Pg 5 |  |  |
|  | **Participant selection** | | |  |  |
|  | 10. Sampling | How were participants selected? e.g., purposive, convenience, consecutive, snowball | Pg 6 |  |  |
|  | 11. Method of approach | How were participants approached? e.g., face-to-face, telephone, mail, email | Pg 6 |  |  |
|  | 12. Sample size | How many participants were in the study? | Pg 10 |  |  |
|  | 13. Non-participation Setting | How many people refused to participate or dropped out? Reasons? | Pg 10 |  |  |
|  | 14. Setting of data collection | Where was the data collected? e.g., home, clinic, workplace | Pg 7 |  |  |
|  | 15. Presence of nonparticipants | Was anyone else present besides the participants and researchers? | N/A |  |  |
|  | 16. Description of sample | What are the important characteristics of the sample? e.g. demographic data, date | Pg 10 |  |  |
|  | **Data collection** | | |  | No |
|  | 17. Interview guide | Were questions, prompts, and guides provided by the authors? Was it pilot tested? | Pg 6 |  |  |
|  | 18. Repeat interviews | Were repeat interviews carried out? If yes, how many? | N/A |  |  |
|  | 19. Audio/visual recording | Did the research use audio or visual recording to collect the data? | Pg 7 |  |  |
|  | 20. Field notes | Were field notes made during and/or after the interview or focus group? | Pg 7 |  |  |
|  | 21. Duration | What was the duration of the interviews or focus group? | Pg 7 |  |  |
|  | 22. Data saturation | Was data saturation discussed? | Pg 7 |  |  |
|  | 23. Transcripts returned | Were transcripts returned to participants for comment and/or correction? | Pg 9 |  |  |
|  | **Domain 3: analysis and findings** | | |  |  |
|  | **Data analysis** | | |  |  |
|  | 24. Number of data coders | How many data coders coded the data? | Pg 8 |  |  |
|  | 25. Description of the coding tree | Did the authors provide a description of the coding tree? | N/A |  |  |
|  | 26. Derivation of themes | Were themes identified in advance or derived from the data? | Pg 8 |  |  |
|  | 27. Software | What software, if applicable, was used to manage the data? | Pg 7 |  |  |
|  | 28. Participant checking | Did participants provide feedback on the findings? | Pg 9 |  |  |
|  | **Reporting** | | |  |  |
|  | 29. Quotations presented | Were participant quotations presented to illustrate the themes/findings? Was each quotation identified? e.g., participant number | Pg 12-21 |  |  |
|  | 30. Data and findings consistent | Was there consistency between the data presented and the findings? | Pg 12-21 |  |  |
|  | 31. Clarity of major themes | Were major themes clearly presented in the findings? | Pg 12-21 |  |  |
|  | 32. Clarity of minor themes | Is there a description of diverse cases or a discussion of minor themes? | Pg 12-21 |  |  |

**Annex B**

**Interview guides**

Based on the RE-AIM and PRISM model: Glasgow RE, Harden SM, Gaglio B, Rabin B, Smith ML, Porter GC, et al. RE-AIM Planning and Evaluation Framework: Adapting to New Science and Practice With a 20-Year Review. Front Public Health. 2019;7:64. doi: 10.3389/fpubh.2019.00064.

**Interview guide for patients**

| Introduction | Thank you for taking part in this interview. We would like to learn more about your experiences with the Energetic programme. I have a list of topics that I wish to address. Feel free to ask questions at any time during the interview. I might make a few notes in case I want to come back to something later.  Could you tell us about your experiences with Energetic? |
| --- | --- |
| Reach | - Could you tell us how you first became involved in the Energetic programme? - How did you register for the programme? - What motivated you to participate in this programme? - Were there any barriers you had to overcome in order to participate? If so, which ones? - What information did you receive beforehand about the programme? |
| Characteristics of the Intervention | - Looking back, how do you reflect on your participation in the programme? - What were your expectations before you started the programme? Were these expectations met? - How would you describe Energetic, and what do you consider to be its most important features? - How did you experience the different modules? - In your view, how does Energetic differ from usual care? - How easy or difficult was it for you to participate in the programme? - How well did the programme match your needs and expectations? - How did you experience the group process? - Could you explain how you experienced the intensity of the programme? - How did you experience the joint approach/cooperation between the occupational therapist and physiotherapist? - How were you communicated with during the programme? What information did you receive? |
| Effectiveness + Implementation and Sustainability Outcomes | - What has participating in the programme brought you? - What changes have you noticed since taking part in the programme? - Are there aspects of the programme you found useful, or not useful? - What are the main outcomes you have achieved by participating in the programme? - Could you give some examples of success stories or important milestones you achieved? - Are there any aspects of the programme that you believe could be improved? What would you like to see done differently? |
| Closing | Thank you for your participation today. |

**Interview guide for therapists**

| Introduction | We are very interested in hearing about your experiences with the implementation of the Energetic programme. We would therefore like to ask you several questions about the implementation process, as well as how you experienced working with the Energetic programme. I have a list of topics that I wish to address. Feel free to ask questions at any time during the interview. I might make a few notes in case I want to come back to something later. We will start at the beginning: recruitment. |
| --- | --- |
| Reach (RE-AIM) | - Can you describe how recruitment was carried out within your practice? - Which strategies were used for recruitment? - What were your experiences with these strategies? What did you think of them? How was this process for you? - What went well, and what would you do differently next time? - In your view, what was the reason that external recruitment did not result in enough patients? - Which factors influenced the recruitment process? - How would you approach this next time? Do you have recommendations for other practices that want to do this? - Are there particular groups of patients who are difficult to reach? What might be the causes? |
| Implementation (RE-AIM) + Implementation and Sustainability Infrastructure (PRISM) | - Could you describe the implementation process of Energetic within your practice? - How did you experience this? - What were the biggest challenges you encountered during implementation? How did you address these challenges? What did you learn from them? - How would you approach this next time? - Are there any specific resources or processes that helped support the implementation? - How were tasks divided between you? Was there a project lead? How did you communicate with each other? - What did you think of this? How would you ideally approach this next time? - What were barriers, and what were facilitating factors? |
| External Environment (PRISM) | - Which collaborations with external partners did you pursue, and how did these go? - How was communication and coordination? - How do you look back on the training sessions and intervision sessions? How did these contact moments contribute to the implementation? Did you miss anything in the guidance? - Which external factors (such as policy, funding, or societal trends) influenced the implementation of the programme? - How does Energetic fit into the financial structure of primary care? How did you make it viable, and what challenges did you face? |
| Characteristics of the Intervention (PRISM) | - What do you consider to be the key characteristics of Energetic? - Where does it differ from regular care? What did you do differently compared to usual practice? - How does Energetic meet the needs of patients? - How well does the programme fit your way of working and your practice? - What aligned well? What aligned less well? - What do you think of the accessibility and usability of the programme and the manual? - Which adaptations were made to better integrate Energetic into your organisation? Organisational adjustments + developed materials? - What were the biggest challenges in tailoring the programme to fit your setting? - How did you experience the use of the webinars? How did you use them and what did you think of them? How did participants respond? What would you do differently next time? - What was it like for you to work at external locations? How did you organise this and how did it go? What would you keep, and what would you change? What recommendations do you have for others? |
| Effectiveness + Implementation and Sustainability Outcomes (PRISM) | - Which measurement instruments did you use during the evaluation? - Can you share anything about patients’ experiences with the programme? - What do you consider to be the most important outcomes achieved? Were you able to evaluate these? - Do you have examples of success stories or important milestones? - Are there improvements we should make to increase effectiveness? |
| Adoption (RE-AIM) + Characteristics of the Implementing Organisation (PRISM) | - How well does Energetic fit within your practice (mission/vision)? Compared to other initiatives? - How does it fit within primary care more broadly? - How did colleagues within the organisation (and externally) respond to the programme? |
| Maintenance (RE-AIM) + Implementation and Sustainability Infrastructure (PRISM) | - What do you think is needed to implement Energetic sustainably? - Are there plans to continue offering the programme? - What are your expectations regarding the time investment for a potential ‘second round’? |
| Additional Points | - Are there any important additions you would like to make? - Which experiences made the greatest impression? What was particularly valuable or especially challenging? |
| Closing | Thank you for your participation today. |
